# Editorial: Cholesterol, inflammation and immunity

**DOI:** 10.3389/fimmu.2025.1696770

**Published:** 2025-09-17

**Authors:** Antonio La Cava, Giuseppe Castaldo

**Affiliations:** ^1^ Dipartimento di Medicina Molecolare e Biotecnologie Mediche, Università di Napoli Federico II, Naples, Italy; ^2^ Department of Medicine, University of California, Los Angeles, Los Angeles, CA, United States; ^3^ CEINGE-Biotecnologie Avanzate Franco Salvatore, Naples, Italy

**Keywords:** cholesterol, inflammation, immunity, HDL - cholesterol, biomarker

Cholesterol metabolism and immune function are tightly interconnected. Cholesterol affects immune cell signaling through the formation of lipid rafts, influences the generation of neutrophil extracellular trap (NET), and modulates both the activity and polarization of macrophages (1). Oxysterols, which are oxygenated derivatives of cholesterol, also regulate various macrophages’ activities and function (2). Conversely, the immune system significantly affects cholesterol synthesis, uptake, and removal (2).

Pathologically, the accumulation of cholesterol in immune cells drives pro-inflammatory responses, establishing a self-sustaining cycle of inflammation. This inflammatory state lowers the circulating levels of high-density lipoprotein (HDL) and impairs cholesterol removal, promoting production of proinflammatory cytokine and favoring the emergence of dysfunctional, proinflammatory HDL that further worsen this imbalance (3, 4). Any disruptions in these intertwined processes can contribute to the initiation and progression of diseases such as atherosclerosis, cardiovascular and metabolic disorders, in addition to autoimmunity, infections, and cancer (5–10). A better understanding of the mechanisms that connect cholesterol metabolism with inflammation and immune responses may enable the identification of novel biomarkers and facilitate the development of new therapies.

This Research Topic reports the identification of new biomarkers associated with dysregulated and/or abnormal interactions between cholesterol metabolism and immune responses.

Carbamylation is a post-translational protein modification common in patients with uremia, and carbamylated proteins have been reported to accumulate in advanced atherosclerotic plaques. Saar-Kovrov et al. found that in plaques from chronic kidney disease patients, carbamylated lysine levels rose with disease stage and inversely correlated with kidney function (assessed as glomerular filtration rate [GFR]). Carbamylation in macrophages associated with the markers CD68, galectin-3 and PLIN2 - a macrophage lipid-associate protein that is involved in lipid accumulation and foam cell formation. Interestingly, macrophage uptake of carbamylated low-density lipoprotein (LDL) promoted foam cell formation similarly to oxidized LDL but without activating peroxisome proliferator-activated receptor (PPAR)γ or causing cytotoxicity, suggesting that carbamylated proteins can contribute to plaque progression in uremia through macrophage foam cell formation via a mechanism that is distinct from oxidized LDL.

Zeng et al. built on the observation that elevated levels of residual cholesterol are known to associate with an increased risk of cardiovascular disease (CVD) and stroke to investigate the association between residual cholesterol and mortality form aneurysmal subarachnoid hemorrhage (aSAH). Residual cholesterol is the cholesterol content in triglyceride (TG)-rich lipoproteins after TGs are removed and mainly includes chylomicron remnants and very low-density lipoproteins (VLDL). Zeng et al.’s finding that residual cholesterol was present at significantly lower levels in non-surviving aSAH patients led the authors to suggest that this newly observed correlation could possibly be exploited to stratify aSAH patients’ mortality risk by measuring residual cholesterol.

Guo and Li utilized the U.S. National Health and Nutrition Examination Survey (NHANES) to study 4,152 participants aged 20–59 years included in the registry in the years 2015–2018. The aim was to investigate possible associations between sarcopenia and a tool that could combine altered lipid metabolism and chronic inflammation. Such tool, the high-sensitivity C-reactive protein (hsCRP)/high-density lipoprotein cholesterol (HDL-C) ratio, was found to positively associate with an increased risk of sarcopenia. While acknowledging the need of additional prospective studies, the authors suggested considering the use of hsCRP/HDL-C to identify individuals at early disease stages or at higher risk of sarcopenia.

Also utilizing the NHANES study on 5,323 adult asthma patients spanning 1999–2018, Tian et al. investigated the mortality risk of asthma patients using another biomarker of systemic inflammation and immune status, the lymphocyte-to-high-density lipoprotein ratio (LHR). The authors reported that an increased LHR associated with a reduced mortality risk (18% for all-cause), 21% for cardiovascular disease (CVD), and 41% for chronic lower respiratory disease (CLRD).

Qin et al. utilized instead serum uric acid to HDL cholesterol ratio (UHR) as an index that could couple inflammation to metabolism, to evaluate associations of this index with *Helicobacter pylori* (*H.p.*) infection. The reported positive correlation between UHR and *H.p.* infection led the authors to suggest possible use of this non-invasive indicator to monitoring *H.p.* infection, proposing it as a new, easy and practical approach to approach a major global health problem.

Han et al. explored the risk factors for hyperuricemia in 714 ethnically homogeneous participants residing in high-altitude areas of the geographical area of Yunnan in China. They found that the greater prevalence of hyperuricemia in this cohort displayed a nearly linear positive correlation with the TG/HDL-c ratio, suggesting that monitoring of TG/HDL-c might benefit patients with hyperuricemia.

Finally, Wang et al. provided an overview of the functions of sphingosine-1-phosphate (S1P) in normal liver physiology, detailing how alterations in the S1P/(S1P receptor (S1PR) signaling axis can contribute to the development of liver diseases. The review examined the pathological mechanisms underlying liver injury, with an emphasis on sepsis. Central to the paper was the exploration of how S1P and S1PR regulate immune responses, bile acid metabolism, and the liver-gut axis during septic liver injury. The authors thoroughly discussed the interplay between S1P/S1PR signaling, hepatic inflammation, and metabolic regulation, as well as the therapeutic potential of modulators targeting this pathway in sepsis-induced liver injury.

## Conclusions

This Research Topic has highlighted the intricate interplay between cholesterol metabolism and immune function, emphasizing how disruptions in this intertwined relationship can contribute to chronic inflammation and various diseases including atherosclerosis and metabolic disorders. Several of the studies included in this topic have reported novel biomarkers that could link lipid metabolism with inflammation and disease risk, offering potential for improved diagnosis and risk stratification of patients. While acknowledging that additional studies are needed to confirm and validate the findings in larger cohorts, biomarker discovery might also represent an informative initial step toward possible future new therapeutic opportunities across a spectrum of inflammatory and metabolic diseases.
